# The Frequency of DNA Mismatch Repair Deficiency Is Very Low in Surgically Resected Lung Carcinoma

**DOI:** 10.3389/fonc.2021.752005

**Published:** 2021-10-06

**Authors:** Naoki Yanagawa, Noriyuki Yamada, Ryo Sugimoto, Mitsumasa Osakabe, Noriyuki Uesugi, Satoshi Shiono, Makoto Endoh, Shin-ya Ogata, Hajime Saito, Makoto Maemondo, Tamotsu Sugai

**Affiliations:** ^1^ Department of Molecular Diagnostic Pathology, Iwate Medical University, Shiwa-gun, Japan; ^2^ Department of Thoracic Surgery, Yamagata Prefectural Central Hospital, Yamagata, Japan; ^3^ Department of Diagnostic Pathology, Yamagata Prefectural Central Hospital, Yamagata, Japan; ^4^ Department of Thoracic Surgery, Iwate Medical University, Shiwa-gun, Japan; ^5^ Department of Pulmonary Medicine, Iwate Medical University, Shiwa-gun, Japan

**Keywords:** lung carcinoma, DNA mismatch repair deficiency, microsatellite instability, PD-L1, immune checkpoint inhibitor

## Abstract

**Introduction:**

DNA mismatch repair (MMR) deficiency leads to changes in the length of nucleotide repeat sequences of tumor DNA. In that situation, DNA replicational errors occur and accumulate during DNA replication. As a result, this mechanism frequently affects the coding regions of oncogenes and tumor suppressor genes and causes carcinogenesis. Recently, DNA MMR deficiency has been recognized as a predictive biomarker for immunotherapy. The aim of this study is to examine the frequency of DNA MMR deficiency and clinicopathological characteristics in surgically resected lung carcinoma (LC) and their correlation.

**Methods:**

A total of 1153 LCs were examined. Tissue microarrays were constructed. The status of MMR deficiency was evaluated by immunohistochemical analysis of MMR protein expression (hMLH1, hMSH2, hMSH6, and hPMS2). Microsatellite instability analysis, *BRAF* mutation, and *MLH1* methylation analysis were performed for cases that showed MMR deficiency.

**Results:**

Only 2 of the 1153 cases (0.17%) showed a loss of hMLH1/hPMS2 protein expression. They also had high levels of microsatellite instability (MSI-H), had neither *MLH1* promoter methylation nor *BRAF* mutation, and were male smokers. Histopathologically, one was a squamous cell carcinoma, and the other was combined small cell carcinoma with squamous cell carcinoma. Regarding PD-L1 protein expression, one had high expression, and the other had none.

**Conclusion:**

The frequency of MMR deficiency was very low in LC. However, our two cases were non-adenocarcinoma and differed from previous studies. Because of its very low frequency, MMR deficiency is not a practical biomarker to predict the effect of immune checkpoint inhibitors in LC.

## Introduction

Lung carcinoma (LC) is the leading cause of cancer-related death in developed countries (1–3). Although multidisciplinary therapy has improved the outcome for LC patients, most of them are diagnosed at an advanced stage, and the 5-year survival rate for LC is still about 18% (1–3). Recently, immunotherapy using immune checkpoint-blocking antibodies targeting programmed death 1/programmed death ligand 1 (PD-1/PD-L1) has improved the outcomes for patients with a variety of malignant tumors (4, 5). PD-1 is expressed on the surface of T cells, while PD-L1 and PD-L2 are expressed on the surface of various cells, including many malignant cells. PD-1 negatively controls its activity by way of interaction with its ligands PD-L1 and PD-L2. This interaction decreases the T-cell activity, resulting in tumor cell avoidance of the immune system (4, 6). Pembrolizumab is one of a humanized monoclonal antibodies against PD-1 that has antitumor activity in advanced non-small cell lung carcinoma. Its therapeutic effect is closely related to PD-L1 expression in cancer cells, and PD-L1 protein expression has been suggested to be a predictive biomarker of the response to immunotherapy (7). Therefore, the status of PD-L1 protein expression has been suggested as a predictive biomarker for immunotherapy.

Meanwhile, DNA mismatch repair (MMR) deficiency has also been recognized as a predictive biomarker for immunotherapy (8–10). DNA MMR deficiency leads to changes in the length of nucleotide repeat sequences of tumor DNA, and a phenomenon called microsatellite instability (MSI) occurs (11). In that situation, DNA replicational errors occur that accumulate during the DNA replication cycle. As a result, this mechanism frequently affects the coding regions of oncogenes and tumor suppressor genes, causing carcinogenesis (12). Le et al. have reported that MMR deficiency predicts the response of various malignant tumors to PD-1 blockade (9). Therefore, it is important to investigate the frequency of DNA MMR deficiency in LC. However, there are few publications regarding the relationship between DNA MMR deficiency and LC, and their relationship is thus inadequately known.

In the present study, we studied the frequency of DNA MMR deficiency and clinicopathological characteristics in surgically resected LC and examined their correlation.

## Patients and Methods

### Patients

A retrospective review of a prospectively maintained surgical database was performed to identify patients who underwent primary LC resection with curative intent from 2008 to 2018 (patients from 2008 to 2014, Yamagata Prefectural Central Hospital; patients from 2015 to 2018, Iwate Medical University). Histopathologic diagnoses had been made in accordance with the eighth edition of the TNM Classification of the Union for International Cancer Control and the 2015 WHO classification (13, 14). Patients were excluded from the current evaluation if they underwent neoadjuvant therapy, underwent incomplete resection, had multiple primary lung cancers, or had incomplete follow-up data. In the end, a total of 1153 patients with primary LC were included. This study was approved by the Institutional Review Board of both Iwate Medical University (reference number: MH2020-163) and Yamagata Prefectural Central Hospital (reference number: 15) and was conducted according to the principles of the Declaration of Helsinki. Written informed consent was waived because this was a retrospective study, the patient data remained anonymous, and an opt-out approach was used.

### Tissue Samples and Tissue Microarray Preparation

A total of 1153 formalin-fixed, paraffin-embedded samples from consecutive resected LC collected from 2008 to 2018 were used for the preparation of tissue microarrays (TMAs). Briefly, we marked one representative tumor area and arrayed a cylindrical 3-mm tissue core from the corresponding paraffin blocks into a recipient block using a tissue arrayer (KIN-2, Azumaya, Japan). Hematoxylin and eosin (HE) staining was used to evaluate tumor cells in each TMA specimen.

### Evaluation of MMR Protein Expression

MMR protein expression was examined by immunohistochemistry (IHC). The TMA blocks were sliced to 4-μm thickness, deparaffinized, and stained for anti-hMLH1 (clone: M1), anti-hMSH2 (clone: G219-1129), anti-hMSH6 (clone: SP93), and anti hPMS2 (clone: A16-4) (Ventana Medical Systems, Inc., Tucson, AZ) on a Benchmark XT system with an automated staining protocol. Tumors showing a total absence of nuclear staining, with the adjacent normal tissue showing the presence of nuclear staining, were regarded as having lost MMR protein expression. Lymphoid cells and stromal cells served as the internal positive staining controls. Two certified pathologists (NY and NU or MO) used a multi-headed microscope to evaluate the slides together and discussed until the consensus was acquired.

### Evaluation of PD-L1 Protein Expression and p53 Protein Expression

PD-L1 protein expression was examined by IHC. The TMA blocks were sliced at 4-μm thickness, deparaffinized, and stained for PD-L1 using the PD-L1 IHC 22C3 pharmDx assay (Agilent Technologies, Inc., Santa Clara, CA, USA) and the Autostainer Link 48 using an automated staining protocol. Two certified pathologists (NY and NU or MO) evaluated the slides. If the cell membrane of the tumor cells was stained, the cells were considered positive for PD-L1 protein expression. The tumor proportion score (TPS) of PD-L1 in tumor cells was defined as the percentage of PD-L1 positive tumor cells among all tumor cells in the TMA tumor sections and was estimated in increments of 5%, except for the 1% value. We classified PD-L1 expression into three patterns based on the TPS as follows: no expression if the TPS was <1%, low expression if the TPS was 1–49%, and high expression if the TPS was ≥50%. We also performed immunohistochemical staining using antibodies against p53 (clone:DO-7, Roche) in the TMAs. The immunohistochemical result was evaluated as follows: staining of p53 protein expression in ≥10% of tumor cells were defined as p53 positivity.

### Microsatellite Instability Analysis

Five NCI markers (BAT-25, BAT-26, D2S123, D5S346, and D17S250) and three additional mononucleotide markers (NR-21, NR-22, and NR-24) were used to determine the presence of MSI in the tumors (15–17). MSI-high (MSI-H) was defined as 2 or more markers being unstable; MSI-low (MSI-L) was defined as 1 marker being unstable; and microsatellite stable (MSS) was defined as the absence of instability.

### *BRAF* Mutation and *MLH1* Methylation Analyses

Mutations in *BRAF* (V600E) genes were examined using a PyroMark Q24 pyrosequencer, as described previously (18, 19). Each reaction contained 1 × PCR buffer, 1.5 mM MgCl_2_, 0.2 mM each dNTP, 5 pmol forward primer, 5 pmol reverse primer (biotinylated), 0.8 U HotStarTaq DNA polymerase (Qiagen), 10 ng template DNA, and dH_2_O to a final volume of 25 μL. Cycling conditions were as follows: 95°C for 15 min; 38 cycles of 95°C for 20 s, 53°C for 30 s, and 72°C for 20 s; and a final extension at 72°C for 5 min, with holding at 8°C. Following amplification, 10 μL biotinylated PCR product was immobilized on streptavidin-coated sepharose beads (Streptavidin Sepharose High Performance; GE Healthcare Bio-Sciences Corp., Piscataway, NJ, USA) and washed in 70% EtOH. The purified biotinylated PCR products were loaded into the PyroMark Q24 (Qiagen) using PyroMark Gold reagents (Qiagen) containing 0.3 lM of the sequencing primer and annealing buffer. Regarding *MLH1* methylation, pyrosequencing for methylation of *MLH1* was performed using a PyroMark Q24 instrument (Qiagen, Valencia, CA, USA), as described in a previous study (20, 21). Briefly, the PCR product (25 μL) was bound to streptavidin Sepharose HP (GE Healthcare, Brøndby, Denmark), purified, washed, denatured in 0.2 M NaOH, and washed again. Before pyrosequencing, 0.3 μM sequencing primer was annealed to the purified single-stranded PCR product by heating to 80°C for 2 min. The primers were designed with the Pyromark Assay Design Software package (Qiagen NV) and included 3 or 4 CpG sites to analyze the methylation of the promoter. The cutoff value for positive methylation was set as 30% of the tumor cells (20).

## Results

### Clinicopathological and Molecular Characteristics

The clinicopathological and molecular characteristics are summarized in **Table 1**. The examined cases comprised 686 males (59.5%) and 467 females (40.5%), with a median age of 69.3 years (range, 23–89). Of the 1153 patients, 709 (61.5%) were smokers. Regarding the pathological stage, 789 patients (68.4%) were classified as stage 0/I, 182 (15.8%) as stage II, 155 (13.4%) as stage III, and 27 (2.3%) as stage IV. Histopathologically, 846 (73.4%) tumors were classified as adenocarcinoma, 242 (21%) as squamous cell carcinoma, and 65 (5.6%) as other histological subtypes. The other subtypes were small cell carcinoma (1.1%), adenosquamous carcinoma (1.1%), large cell carcinoma (0.9%), pleomorphic carcinoma (0.8%), carcinoid (0.7%), large cell neuroendocrine carcinoma (0.6%), combined small cell carcinoma (0.2%), adenoid cystic carcinoma (0.2%), and mucoepidermoid carcinoma (0.1%). PD-L1 protein expression was as follows: none, 933 (80.9%); low, 106 (9.2%); and high, 114 (9.9%). Finally, p53 protein expression was positive in 427 (37%).

**Table 1:** Clinicopathological and molecular characteristics in examined Lung Carcinoma.

Characteristics	N = 1153
Sex	
Male	686 (59.5%)
Female	467 (40.5%)
Age (years)	23-89 (average 69.3)
Smoking	
No	444 (38.5%)
Yes	709 (61.5%)
Stage	
0-I	789 (68.4%)
II	182 (15.8%)
III	155 (13.4%)
IV	27 (2.3%)
Histopathology	
Adenocarcinoma	846 (73.4%)
Squamous cell carcinoma	242 (21.0%)
Others	65 (5.6%)
Small cell carcinoma	13 (1.1%)
Adenosquamous carcinoma	13 (1.1%)
Large cell carcinoma	10 (0.9%)
Pleomorphic carcinoma	9 (0.8%)
Carcinoid	8 (0.7%)
Large cell neuroendocrine carcinoma	7 (0.6%)
Combined small cell carcinoma	2 (0.2%)
Adenoid cystic carcinoma	2 (0.2%)
Mucoepidermoid carcinoma	1 (0.1%)
PD-L1 protein expression	
No expression	933 (80.9%)
Low expression	106 (9.2%)
High expression	114 (9.9%)
p53 protein expression	
Negative expression	726 (63.0%)
Positive expression	427 (37.0%)

### The Frequency of DNA MMR Deficiency and Its Correlation With Clinicopathological and Molecular Characteristics

**Figure 1:**
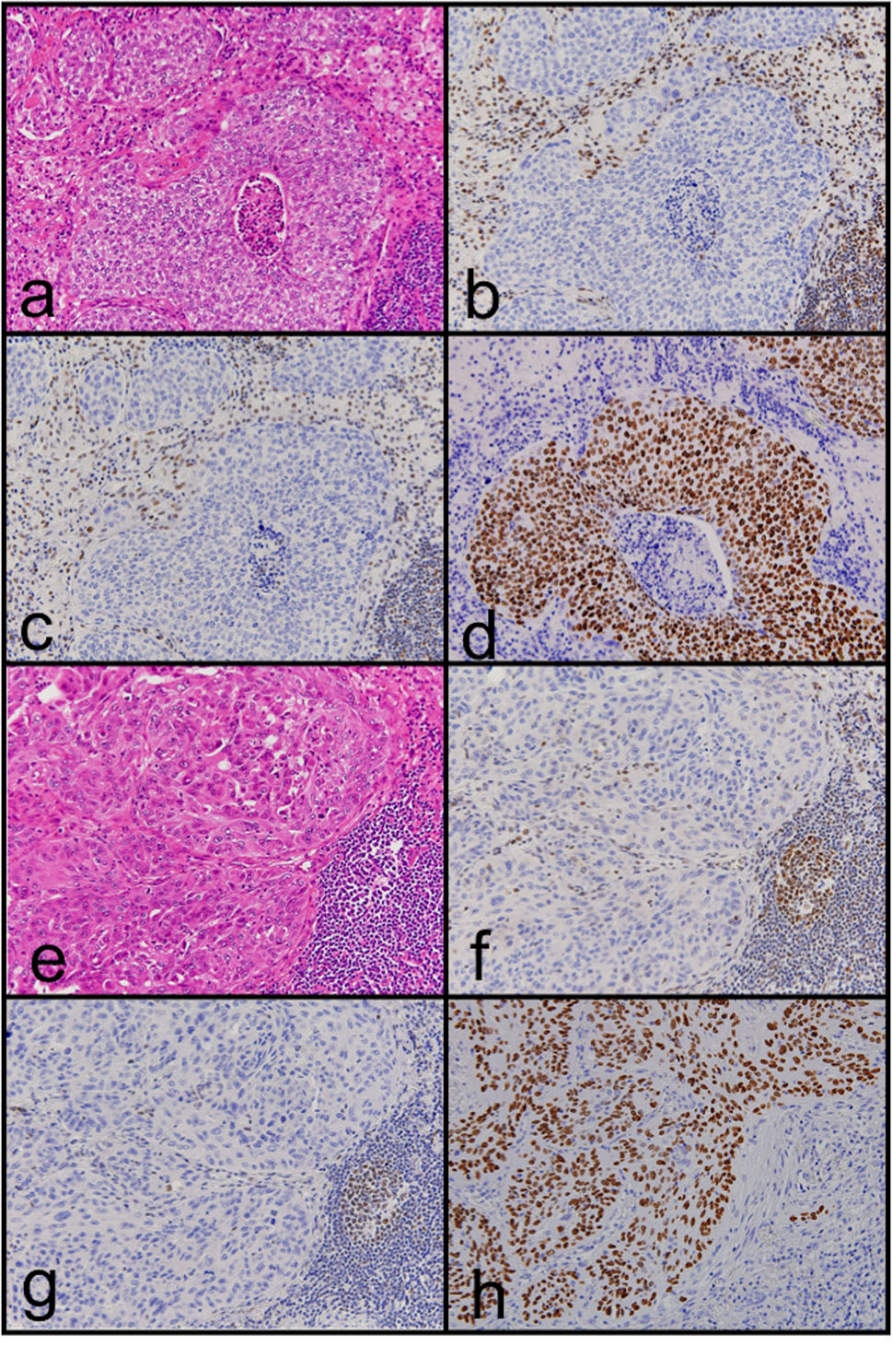
Representative images of hematoxylin and eosin staining **(A, E)** and immunohistochemical staining of hMLH1 **(B, F)** and hPMS2 **(C, G)** in MMR deficiency lung carcinoma. Loss of hMLH1 and hPMS2 expression can be observed. In addition, p40 protein expression can be observed **(D, H)**. Case 1 is **(A)** to **(D)**; case 2 is **(E)** to **(H)**.

**Figure 2:**
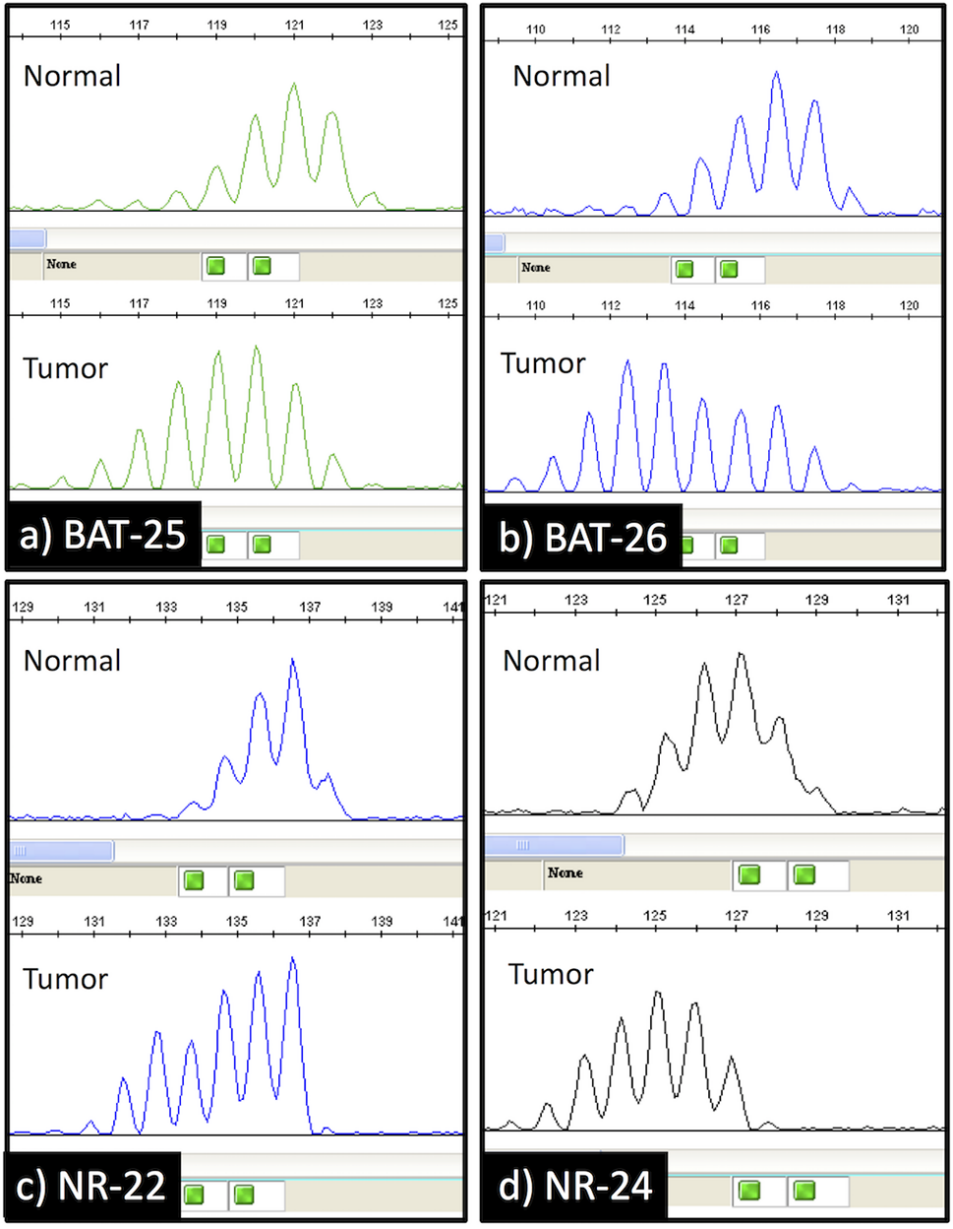
Capillary electrophoresis results of the microsatellite instability analysis. The shift in the size (bases) of the amplification products in the tumor specimen compared to the normal lung tissue specimen was observed at 4 mononucleotide repeat loci (BAT-25, BAT-26, NR-22, and NR-24).

Loss of MMR protein expression was only observed in 2 of 1153 cases (0.17%). Both of them showed loss of hMLH1 and hPMS2 protein expression (**Figure 1**). They also had MSI-H (**Figure 2**). They had neither *MLH1* promoter methylation nor *BRAF* mutation. Briefly, both were males and smokers. One was pathological stage I and the other was stage II. One is alive and the other was died in 6 months after surgical resection because of recurrence. One had a history of gastric carcinoma, and the other had a history of colon carcinoma. Neither had any remarkable family history and not adapted to criteria about Lynch syndrome (22, 23). Histopathologically, one was a squamous cell carcinoma, and the other was a combined small cell carcinoma with squamous cell carcinoma. The latter showed loss of hMLH1/hPMS2 protein expression together with the presence of MSI-H only in the squamous cell carcinoma area. We also examined immunohistochemistry for our cases. We used four antibodies as follows: TTF-1 (8G7G3/1, Dako, Glostrup, Denmark), Napsin A (IP64, Nichirei, Tokyo, Japan), p40 (BC28, Biocare Medical, Pacheco, CA, USA) and CK5/6 (D5/16 B4, Dako, Glostrup, Denmark). p40 and CK 5/6 were both positive (**Figure 1**), on the other hand, TTF-1 and Napsin A were both negative. One had high expression of PD-L1 protein while the other had none, and both showed overexpression of p53 protein.

## Discussion

In the present study of 1153 lung carcinoma cases, only 2 (0.17%) showed MMR deficiency. The occurrence of MMR deficiency in lung carcinoma has been reported in only a few large-scale studies. Warth et al. detected MSI-H in 4/480 (0.8%) cases of pulmonary adenocarcinoma (24); Takamochi et al. detected MSI in only 1/341 (0.3%) cases of lung adenocarcinoma (25); and Vanderwalde et al. detected MSI-H in 12/1868 (0.6%) cases of non-small cell lung carcinoma (26). The frequency of MMR deficiency was from 0.3% to 0.8%. Our frequency of 0.17% was a little lower than the 0.3% to 0.8% of these previous studies. Differences in the method of detection may underlie the small differences among these studies. Results from these previous studies are shown together with our results in **Table 2**. Note that all MSI-H patients for whom smoking history was reported were smokers. MMR-deficient tumors have been reported to show a good response to pembrolizumab in some types of carcinoma that were reported to have a higher nonsynonymous mutation burden (10, 27, 28). Tumor mutation burden is used as a biomarker to predict a good response to immunotherapy because tumors with a high mutation burden present neoepitopes that can behave as neoantigens (29). Tobacco smoke carcinogens induce DNA damage, p53 mutation, and nonsynonymous mutation burden (27, 30, 31), and the molecular signature with smoking is significantly correlated with nonsynonymous mutation burden (25, 27). Higher nonsynonymous mutation burden in tumors is associated with improved objective response, durable clinical benefit, and progression-free survival after treatment with immune checkpoint inhibitor (32). Thus, we consider that the tumor of our MMR-deficient LC patient showed mutational signatures associated with both smoking and DNA MMR deficiency.

**Table 2:** Large-scale studies about MMR deficiency in Lung Carcinoma.

Study	Examined cases	Frequency of MSI-H	Sex	Histopathology	Stage	Smoking history	Other carcinoma	PD-L1 expression	p53 expression
Warth et al. (24)	480 (ADC)	4 (0.8%)	Female (1), Male (3)	ADC	All of stage I	All	Esophagus (1), Kidney (1), Pancreas (1)	ND	ND
Takamochi et al. (25)	341 (ADC)	1 (0.3%)	Male (1)	ADC	ND	All	No history	No	ND
Vanderwalde et al. (26)	1868 (NSCLC)	12 (0.6%)	ND	ND	ND	ND	ND	ND	ND
Yanagawa et al. (our study, 2021)	1153 (Lung carcinoma)	2 (0.17%)	Male (2)	SQCC (1) and combined SCLC (1)*	stage I (1), stage II (1)	All	Stomach (1), colon (1)	No (1), High (1)	positive (2)

MMR, mismatch repair; ADC, adenocarcinoma; NSCLC, non-small cell lung carcinoma; MSI, microsatellite instability; SQCC, squamous cell carcinoma; SCLC, small cell lung carcinoma; ND, not do.

As to histopathological subtypes, our cases were not adenocarcinomas. Only adenocarcinomas were examined in the two previous studies (24, 25), while another study did not report histological subtypes (26). MSI is frequently observed in endometrial carcinoma, gastric and colorectal carcinoma, and small intestine malignancies, and most of these are considered to be adenocarcinoma (26). On the other hand, MSI was not detected in head and neck squamous carcinoma and esophageal carcinoma, which are considered to be squamous cell carcinomas (26). Therefore, we think our cases were rare.

In sporadic colon and gastric carcinoma, *BRAF* mutation and the epigenetic inactivation of *MLH1* expression by promoter methylation result in MMR deficiency (17, 33). Our cases showed loss of hMLH1/hPMS2 protein expression and MSI-H, and therefore we examined *BRAF* mutation and *MLH1* promoter methylation. However, neither *BRAF* mutation nor *MLH1* promotor methylation was detected. Besides, our cases did not fit with criteria of Lynch syndrome. A majority of Lynch syndrome patients have germline mutations in 1 of the 4 MMR genes: *MLH1*, *MSH2*, *MSH6*, and *PMS2* (8). These mutations induce MSI and the patients with Lynch syndrome have a high tendency to suffer from multiple cancers such as colorectal, uterine, ovarian, stomach, small bowel, pancreatic, kidney, and brain. However, their risk of lung cancer is considered to be the same as that of the general population (34). It is reported that somatic mutation of MMR genes also results in MMR deficiency in some carcinomas (35). Takamochi et al. have reported that somatic *MLH1* gene mutation of lung adenocarcinoma is correlated with MSI (25). Our cases may have somatic *MLH1* gene mutation; further examination will be needed.

This study has some limitations. First, we used TMAs rather than whole tissue sections, possibly missing changes in consequence of heterogeneous immunohistochemical staining. Secondly, we assessed the deficiency of MMR protein by using IHC prior to MSI analysis, whereas previous studies did MSI analysis in advance of determining the deficiency of MMR protein using IHC. Thirdly, we could not examine somatic *MLH1* gene mutation.

## Conclusion

The frequency of MMR deficiency was very low in LC. Interestingly, our cases were not adenocarcinomas and were thus different from those of previous studies. Because its frequency is so very low, MMR deficiency does not appear to be a practical biomarker to predict the effect of immune checkpoint inhibitors in LC.

## Data Availability Statement

The original contributions presented in the study are included in the article/supplementary material. Further inquiries can be directed to the corresponding author.

## Ethics Statement

This study was approved by the Institutional Review Board of both Iwate Medical University (MH2020-163) and Yamagata Prefectural Central Hospital (15) and was conducted according to the principles of the Declaration of Helsinki. Written informed consent was waived because this was a retrospective study, the patient data remained anonymous, and an opt-out approach was used.

## Author Contributions

Conception and writing of the manuscript: NaY and TS. Collection of the clinical data: NaY, SS, ME, MM, and HS. Pathological diagnosis and immunohistochemical analyses: NaY, S-yO, NoY, RS, MO, and NU. Collection of the samples for the molecular analyses: NaY. All authors contributed to the article and approved the submitted version.

## Conflict of Interest

The authors declare that the research was conducted in the absence of any commercial or financial relationships that could be construed as a potential conflict of interest.

## Publisher’s Note

All claims expressed in this article are solely those of the authors and do not necessarily represent those of their affiliated organizations, or those of the publisher, the editors and the reviewers. Any product that may be evaluated in this article, or claim that may be made by its manufacturer, is not guaranteed or endorsed by the publisher.
